# Oxygen Therapy for Intracranial Hemorrhage

**DOI:** 10.1002/cns.70806

**Published:** 2026-02-24

**Authors:** Qiqi Shi, Song Han, Xiangyu Li, Jingwei Guan, Yanli Duan, Zhangyuan Liao, Zhiying Chen, Weili Li, Ran Meng, Ming Zou, Jiayue Ding

**Affiliations:** ^1^ Department of Neurology Tianjin Medical University General Hospital Tianjin China; ^2^ Department of Neurology Tianjin Huanhu Hospital Tianjin China; ^3^ Department of Neurology Affiliated Hospital of Jiujiang University Jiujiang Jiangxi China; ^4^ Department of Neurology, WeiFang People's Hospital Shandong Second Medical Univeristy Weifang Shandong China; ^5^ Department of Neurology, Xuanwu Hospital Capital Medical University Beijing China

**Keywords:** hyperbaric hyperoxia, intracranial hemorrhage, normobaric hyperoxia, oxygen therapy

## Abstract

**Aims:**

Intracranial hemorrhage (ICH) is a severe cerebrovascular disease with a high mortality rate that impairs patient well‐being and quality of life. Oxygen therapies, including hyperbaric hyperoxia (HBO) and normobaric hyperoxia (NBO), have attracted widespread attention as potential adjuvant treatments because of their neuroprotective effects. This review aimed to summarize the current literature addressing the neuroprotective mechanisms of oxygen therapy in ICH, as well as its effectiveness and safety in patients with ICH.

**Methods:**

We systematically searched multiple literature databases including PubMed, Embase, and Cochrane for publications containing specified keywords and published prior to November 2025. The references were thoroughly reviewed to identify other articles that may have been missed in our search.

**Results:**

A total of 38 articles were included in this study. Among them, 20 mainly studied the mechanism of oxygen therapy after ICH, eight mainly investigated the effects of oxygen therapy in patients with ICH, and 10 primarily analyzed the safety of oxygen therapy in patients with ICH. The experimental results showed that the treatment mechanism of HBO mainly involves reducing cerebral vasospasm, promoting angiogenesis, inhibiting inflammatory responses, and improving aerobic energy metabolism, whereas NBO mainly protects the blood–brain barrier (BBB), reduces cerebral edema and hemispheric swelling, mitigates acute inflammation, inhibits oxidative stress and neuronal cell death, and enhances aerobic metabolism. Clinical trials have shown that oxygen therapy can improve neurological function recovery and long‐term prognosis in patients with ICH, as reflected by better scores in some indicators and lower mortality rates; however, oxygen therapy also has controversial and potential risks. Excessive oxygen supply may lead to adverse reactions, and hyperoxia may negatively impact patients with ICH. An appropriate treatment plan should be formulated for the clinical application of oxygen therapy.

**Conclusion:**

Oxygen therapy shows potential in ICH treatment through multiple mechanisms; however, its safety and optimal regimen require further large‐scale randomized controlled trials to balance the benefits and risks and optimize its application strategies.

## Graphical abstract

Oxygen therapies confer neuroprotective effects on patients with intracranial hemorrhage through mechanisms such as anti‐inflammation, reduction of cerebral edema and neuronal death, and enhancement of aerobic metabolism. Clinical evidence suggests that oxygen therapy may improve neurological recovery and decrease mortality in patients with intracranial hemorrhage; however, excessive oxygen administration poses potential risks.

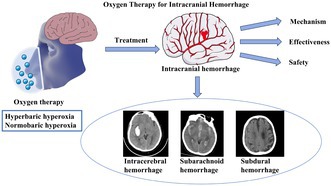

## 1 Introduction

Intracranial hemorrhage (ICH) is an intractable and life‐threatening stroke subtype that accounts for approximately 15%–20% of stroke cases in China. Therapeutic strategies for ICH deserve more attention because of their high death and disability rates [1]. Although neurosurgery (such as hematoma aspiration and decompressive craniectomy) and drugs (such as dehydrants and antihypertensive agents) are widely used in clinical settings, their safety and efficacy require further improvement [2]. Therefore, a new adjuvant strategy for ICH treatment is required to achieve a more satisfactory prognosis. In this context, oxygen therapies, including hyperbaric hyperoxia (HBO) and normobaric hyperoxia (NBO), have received widespread attention because of their potential neuroprotective effects. HBO is a treatment method in which a patient intermittently inhales 100% pure oxygen in a high‐pressure chamber at a pressure greater than sea level, that is, greater than 1 absolute atmosphere (ATA) [3]. NBO is a treatment method that provides high‐concentration oxygen through a mask or nasal cannula at sea level (1 ATA) to increase the oxygen partial pressure. The oxygen concentration fraction was > 30% [4, 5]. Neuroprotective mechanisms of oxygen therapy in ischemic stroke have been extensively investigated [6]. Both HBO and NBO can increase oxygen pressure surrounding the infarction, thereby protecting the penumbra and retarding infarction extension [5, 7]. In theory, the perihematoma in ICH is similar to the penumbra in ischemic stroke, both of which are ischemic–hypoxic. Therefore, oxygen therapy may be a promising therapeutic strategy for ICH. Oxygen therapy can also improve the microenvironment surrounding the hematoma through reducing cerebral edema, inhibiting microglial hyperactivation, and promoting angiogenesis [8, 9, 10, 11]. However, the safety, efficacy, and mechanisms of action of oxygen therapy remain unclear. Therefore, this review aimed to summarize the current evidence and provide a comprehensive understanding of the neuroprotective mechanisms and optimized management strategies for oxygen therapy in patients with ICH. Compared with previous reviews on oxygen therapy in stroke, this review includes recent studies, such as the study on the mechanism by which oxygen therapy inhibits subarachnoid hemorrhage (SAH)‐induced vasospasm changes [12]. In addition, this review not only systematically summarizes the mechanisms of oxygen therapy in ICH but also integrates mechanistic and safety perspectives. This elucidates the therapeutic potential and risks associated with oxygen therapy for ICH.

## 2 Methods

We searched multiple literature databases, including PubMed, Embase, and Cochrane, for publications with specific keywords, “intracranial hemorrhage” or “intracerebral hemorrhage” or “cerebral hemorrhage” and “oxygen therapy” or “normobaric oxygen/normobaric hyperoxia/eubaric oxygen/eubaric hyperoxia” or “hyperbaric oxygen/hyperbaric hyperoxia,” which were published prior to November 2025. The references were thoroughly reviewed to identify other articles that may have been missed in our search.

**FIGURE 1:**
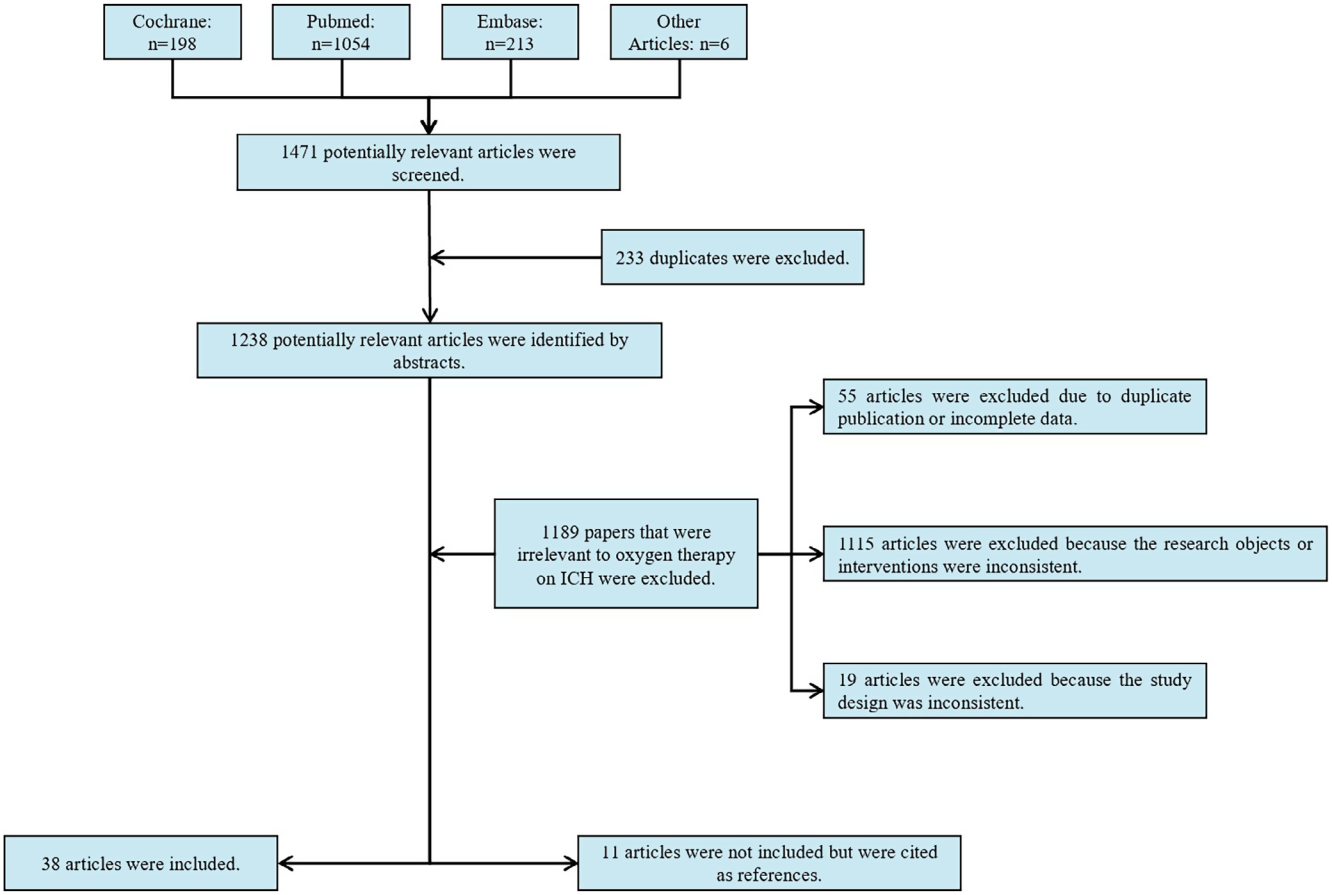
Selection flowchart for this review.

The inclusion criteria are set as follows: (a) patients with a confirmed diagnosis of ICH (including cerebral parenchymal hemorrhage, SAH, epidural, subdural, and intraventricular hemorrhages) or hemorrhagic transformation, regardless of age, sex, ethnicity, or disease stage; or animal models of ICH, regardless of species or strain; (b) the experimental group received the target intervention (HBO or NBO therapy), and the control group received a comparator intervention; (c) comparative studies (including randomized controlled trials, cohort studies, and case–control studies) and basic research; and (d) published in English. Articles that are duplicate publications or have incomplete data were excluded. Additionally, articles related to oxygen therapy for ICH, which can serve as a basis for extrapolation but has inconsistent research subjects or interventions or inappropriate study design such as case reports, comments, reviews, systematic reviews and meta‐analyses, were not included but cited as references (Figure 1). Finally, 38 articles were included in this study.

By reading the abstracts, the included articles were classified into the following categories based on their research purposes and conclusions: (a) investigated one or more mechanisms of oxygen therapy for ICH; (b) examined the clinical therapeutic effects of oxygen therapy for ICH; and (c) studied the impact of hyperoxia on patients with ICH and investigated the oxygen therapy regimens suitable for patients with ICH. Among them, 20 mainly studied the mechanism of oxygen therapy for ICH, eight mainly studied the effects of oxygen therapy in patients with ICH, and 10 mainly studied the safety of oxygen therapy in patients with ICH (Figure 1).

## 3 Mechanisms of Oxygen Therapy in ICH

ICH is categorized based on its anatomical site, including cerebral parenchymal hemorrhage, SAH, epidural, subdural, and intraventricular hemorrhages [13, 14]. The acute phase of ICH can be managed with antihypertensive drugs, hemostatic drugs, and surgery [15]. Neuroprotective agents, such as edaravone, are often used in neuroprotection and rehabilitation therapy [16, 17]. Compared to traditional treatment strategies, oxygen therapy is a convenient, noninvasive, and safe adjuvant method with prominent neuroprotective effects against ICH through various mechanisms [2, 18, 19].

### 3.1 Mechanisms of HBO Treating ICH

The neuroprotective mechanisms of HBO in ICH can be summarized as the reduction of cerebral vasospasm, promotion of angiogenesis, inhibition of inflammatory responses, and improvement of aerobic energy metabolism.

The application of HBO in the treatment of ICH has been extensively studied. Most experimental studies have identified the neuroprotective effects of HBO in animals with ICH, and its mechanisms of action have been thoroughly investigated. The acute phase of ICH is critical for HBO treatment. Lekic et al. [20] have reported that HBO could reduce brain tissue damage and normalize brain atrophy, suggesting promising application prospects for early intervention. Peripheral blood flow decreased at the beginning of HBO exposure, followed by a gradual increase, maintenance of this level, or increase for at least 10 min after exposure compared to the baseline. Additionally, transcutaneous oxygen pressure levels also increase throughout the treatment profile [21]. Thus, peripheral blood flow increases after prolonged HBO exposure. Qin et al. [22] have demonstrated that HBO preconditioning could reduce perihematoma edema and improve local cerebral blood flow, which is associated with the suppression of inflammation and maintenance of blood–brain barrier (BBB) integrity. HBO treatment also suppresses oxidative stress and reduces cell injury in the cortex and hippocampus [23]. These neuroprotective effects of HBO are translated into functional neurological improvements.

HBO significantly attenuates SAH‐induced vasospasm changes, displayed as an increased vasospasm index and arterial wall thickness [24]. Liu et al. [12] have reported that the mammalian target of rapamycin (mTOR) was significantly increased after HBO administration, which participates in several signaling pathways, including cell growth and proliferation, and is negatively correlated with cerebral vasospasm in SAH. Furthermore, HBO treatment upregulates the expression of B‐cell lymphoma‐2 (Bcl‐2) and suppresses the formation of Bcl‐2‐related X protein (Bax) dimers by regulating the phosphatidylinositol 3‐kinase (PI3K)/protein kinase B (Akt)/mTOR pathway. Bcl‐2 is an anti‐apoptotic protein, whereas Bax is a pro‐apoptotic protein. The promotion of Bcl‐2 expression and inhibition of Bax expression alleviate apoptosis, thereby relieving cerebral vasospasm [12]. Through this mechanism, HBO significantly inhibits delayed cerebral vasospasm. Through these complex effects on SAH‐related pathophysiology, HBO improves cerebral blood flow, reduces intracranial pressure, and increases cerebral perfusion pressure [25, 26].

HBO can promote angiogenesis after intracerebral hemorrhage, as demonstrated by the appearance of numerous vessel‐like structures and generation of microvessels following HBO administration. This is because HBO can significantly increase the expression of hypoxia‐inducing factor 1‐α (HIF‐1α) during the repair and regeneration phase after intracerebral hemorrhage (several days to weeks after intracerebral hemorrhage). HIF‐1α regulates vascular endothelial growth factor (VEGF) expression, which can promote angiogenesis in intracerebral hemorrhage rats. Moreover, HBO enhances the expression of proliferative cell nuclear antigen (PCNA) and von Willebrand factor (vWF) in intracerebral hemorrhage rats [8]. PCNA and vWF are associated with vascular structural integrity and cell proliferation. Therefore, HBO can repair and reconstruct the vascular wall in damaged brain tissue.

HBO attenuates neuroinflammation after intracerebral hemorrhage in rats by regulating microglial characteristics and reducing pro‐inflammatory cytokine levels. The growth of ionized calcium binding adapter molecule 1‐positive microglia was lower in the HBO group than that in the intracerebral hemorrhage group [27]. The intracerebral hemorrhage‐induced levels of proinflammatory cytokine, including tumor necrosis factor‐α and interleukin‐1β, and the phosphorylation of c‐Jun amino terminal kinase (JNK) and signal transduction and activator of transcription 1 (STAT1) were lower in the HBO rats. Pro‐inflammatory cytokines are strongly associated with activated microglia, and controlling microglial activation can contribute to the regulation of brain inflammation. STAT1 is a member of the STAT family and a downstream regulatory factor of the JNK pathway, which is involved in regulating microglial cell activation and M1 polarization. In rats with intracerebral hemorrhage, HBO attenuates M1 microglia/macrophage polarization through the JNK/STAT pathway [10]. Additionally, HBO improved brain inflammation in intracerebral hemorrhage rats by silencing microRNA‐204‐5p‐targeted chloride channel protein 3 (CLCN3), which is a key neuronal protein involved in the regulation of microglial polarization [28].

HBO can profoundly improve aerobic energy metabolism [29], thereby preventing the irreversible dysfunction of neurons and vascular endothelial cells. A study conducted in patients with acute subdural hematoma has demonstrated that HBO treatment might promote cerebral circulation and metabolism coupling. Increased lactate production indicates an anaerobic metabolic status caused by the lack of oxygen and mitochondrial damage. Decreased lactate levels after HBO treatment may indicate that HBO improves aerobic metabolism [30]. One reason is that HBO decreases the expression and activity of nicotinamide adenine dinucleotide phosphate oxidase (NOX). Neuronal NOX enzymatic activity increased 24 h after SAH, accompanied by elevated gp91^phox^ mRNA expression levels. NOX is a membrane‐bound enzyme that may produce superoxide anions directed toward the cell interior in non‐phagocytic cells, thus causing oxidative stress. The catalytic subunit gp91^phox^ is one such component. HBO reduces gp91^phox^ mRNA expression 24 h after SAH and suppresses oxidative stress by decreasing NOX enzymatic activity [23]. Additionally, HBO reduces lipid peroxidation following SAH. This inhibitory effect may be mediated by the restoration of high‐energy phosphates or rapid activation of antioxidant defenses. HBO can prevent rebleeding by activating the ATP/nicotinamide adenine dinucleotide (NAD^+^)/silent mating type information regulation 2 homolog 1 (Sirt 1) pathway under hyperglycemic conditions. HBO positively modulates nicotinamide phosphoribosyltransferase and upregulates NAD^+^ levels by increasing ATP levels. NAD^+^ plays an important role in energy balance [31]. Compared to traditional intracerebral hemorrhage animals, high‐altitude intracerebral hemorrhage animals exhibited more severe neurological deficits, brain edema, and neuronal injury. HBO can significantly relieve brain injury caused by high‐altitude intracerebral hemorrhage by increasing partial brain tissue oxygen pressure and decreasing glutamate levels, which promote aerobic metabolism and suppress neurotoxicity [32].

The activation of p44/42 mitogen‐activated protein kinases (MAPK) may play a key role in HBO‐induced protection against hemorrhagic brain edema, and this protective effect may be partially caused by the upregulation of heat shock proteins [22]. Ribosomal protein S6 kinases (RPS6K) are activated by HBO. The major target of activated RPS6K is ribosomal protein S6, which is a major regulator of protein synthesis [9]. Increasing new protein synthesis reduces brain edema by reducing the delay in protection following ICH. Additionally, aquaporin 4 (AQP4) in the brain is the structural basis of water transport between the cerebrospinal fluid and cells, and AQP4 levels can affect the activity of K^+^ channels on the cell membrane, which disrupts the balance of K^+^ and water and changes the osmotic pressure, leading to further exudation of plasma proteins and water. HBO decreases AQP4 expression in the brain tissue of rabbits with cerebral hemorrhage and suppresses the progression of brain edema [33]. Early HBO therapy after intracerebral hemorrhage can reduce cerebral edema and protect the BBB by downregulating HIF‐1α expression [11]. This is not contradictory to the previous finding that HBO can increase HIF‐1α expression during the angiogenesis phase after intracerebral hemorrhage, as they occur in different disease stages and HIF‐1α exerts distinct roles [8, 11].

### 3.2 Mechanisms of NBO Treating ICH

NBO can protect neurological function after ICH through multiple mechanisms. Several animal studies have confirmed these mechanisms. However, studies comparing NBO treatment for ICH compared with HBO treatment are few. NBO reduces BBB damage and hemispheric swelling, which are critical contributors to secondary brain injury [34, 35]. NBO therapy with oxygen delivered at 90% concentration significantly reduced water content and cellular apoptosis in the perihematoma, suggesting an amelioration of brain edema and neuronal injury, and likely contributed to a better neurologic outcome [36]. Additionally, it mitigates acute inflammation after ICH, thereby inhibiting the oxidative stress response and neuronal cell death [37]. Moreover, extrapolation from the results of a study in patients with ischemic stroke suggests that NBO enhances aerobic metabolism in the brain, which is essential for preserving neuronal integrity and promoting functional recovery [38].

Tight junction proteins, including claudin‐5 and occludin, seal the gaps between endothelial cells, prevent paracellular diffusion between endothelial cells, and are important structural components that maintain BBB integrity. The activation and proteolytic activity of matrix metalloproteinases (MMPs), particularly MMP‐9, are key factors in the proteolytic disruption of the basal lamina and tight junctions of the BBB. NBO can inhibit MMP‐9 activity in the ischemic brain, thereby preventing occludin degradation [11, 39]. Additionally, early oxygen therapies reduced the posthemorrhagic expression of HIF‐1α. Another potential mechanism of BBB degradation may be upregulation of VEGF, a major HIF‐1α downstream protein, which enhances MMP activity and thereby increases BBB permeability. HIF‐1α attenuation is involved in the BBB‐protective effects of early oxygen therapy [11, 36].

NBO has some beneficial effects on brain metabolism following ICH. NBO reduces brain lactate and preserves N‐acetyl‐aspartate, which is a marker of neuronal integrity and mitochondrial function in patients, suggesting that NBO improves aerobic metabolism and restores mitochondrial function [38]. Complement component 3 (C3) has been implicated as a driver of acute inflammation after ICH and can exacerbate the oxidative stress response and neuronal cell death. Hemorrhagic injury resulted in the high plasma C3 levels in patients with ICH, and the plasma C3 levels were closely related to hemorrhagic severity and clinical outcomes after ICH. This finding was also observed in a mice model. Microglia mediate synaptic pruning following ICH via the complement system. NBO alleviates brain injury and plays a neuroprotective role following ICH by reducing C3 levels in microglia, inhibiting complement system activation, and alleviating microglia‐mediated synaptic pruning [37].

**FIGURE 2:**
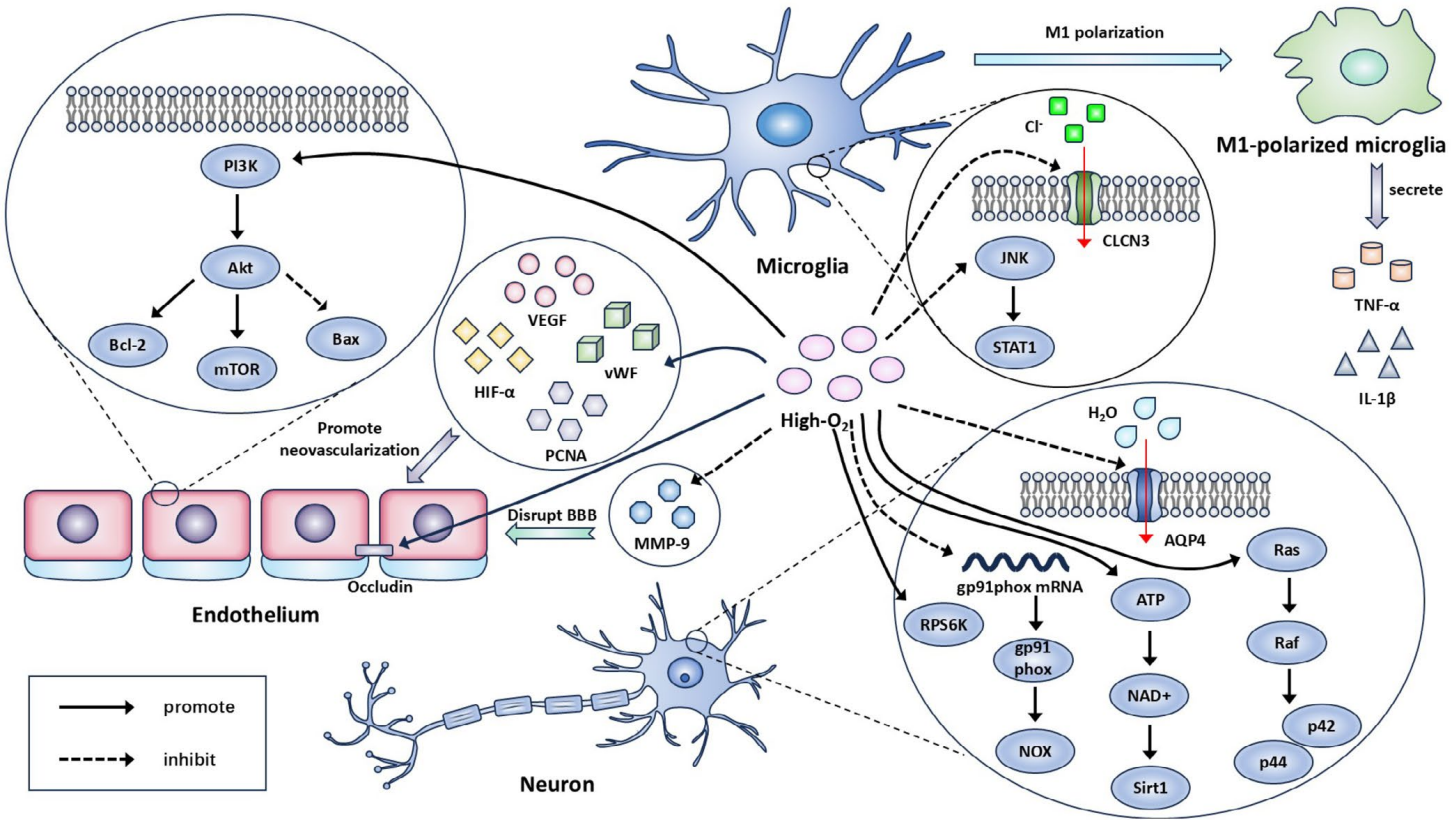
Mechanism of oxygen therapy after ICH. Oxygen therapy exerts therapeutic effects after ICH through multiple mechanisms: (1) regulating the PI3K/Akt/mTOR pathway, up‐regulating the expression of Bcl‐2 and suppressing the formation of Bax dimer, reducing cell apoptosis, inhibiting the occurrence of delayed cerebral vasospasm; (2) increasing angiogenesis‐related factors (VEGF, vWF, HIF‐α and PCNA are involved in the regulation of the angiogenic process), promoting the formation of new blood vessels; (3) regulating the JNK/STAT pathway and reducing the CLCN 3, attenuating M1 microglia/macrophage polarization, reducing the level of the inflammatory factors (TNF‐α and IL‐1β), reducing the inflammatory response; (4) inhibiting gp91^phox^ mRNA expression, reducing the production of gp91^phox^ proteins, restraining NOX, controlling the ROS level, avoiding cell damage caused by oxidative stress; (5) inhibiting the degradation of occludin, suppressing the upregulation of MMP‐9, protecting the BBB; (6) activating the ATP/NAD^+^/Sirt 1 signaling pathway, reducing the risk of bleeding transformation under hyperglycemia conditions, promoting aerobic metabolism; (7) activating the RPS6K and p44/42 MAPK pathways and reducing AQP4, reducing brain edema after ICH.

In summary, oxygen therapy treats ICH through the following pathways (Figure 2): (1) PI3K/Akt/mTOR pathway—regulation of the PI3K/Akt/mTOR pathway, reduce cell apoptosis and inhibit the occurrence of delayed cerebral vasospasm; (2) angiogenesis‐related factors—increasing angiogenesis‐related factors, promote the formation of new blood vessels; (3) microglial cell polarization—regulation of the JNK/STAT pathway and reduction of the CLCN3, attenuate M1 microglia/macrophage polarization, reduce the inflammatory response; (4) oxidative stress—reduction of the production of gp91^phox^ proteins, reduce oxidative stress; (5) BBB—inhibition of the degradation of occludin, protect the BBB; (6) aerobic metabolism—activation of the ATP/NAD^+^/Sirt 1 signaling pathway, reduce the risk of bleeding transformation under hyperglycemia conditions; and (7) brain edema—activation of the RPS6K and p44/42 MAPK pathways and reduction of AQP4, reduce brain edema after ICH. Additionally, we integrated overlapping mechanisms between HBO and NBO. The comparison of these mechanisms is presented in Table 1.

**TABLE 1:** Mechanisms of oxygen therapy in intracranial hemorrhage.

Mechanism	HBO and NBO overlapping mechanisms	HBO‐specific mechanisms	NBO‐specific mechanisms	Study
Attenuation of vasospasm	—	HBO attenuates vasospasm index and arterial wall thickness	—	Celik, 2014 [24] Liu, 2024 [12]
	—	Pathway: activation of PI3K/Akt/mTOR pathway	—	Liu, 2024 [12]
Promotion of angiogenesis	—	HBO promotes formation of numerous perihematomal vessel‐like structures and microvessels	—	Peng, 2014 [8]
	—	Pathway: upregulation of HIF1‐α, VEGF, PCNA and vWF (several days to weeks after intracerebral hemorrhage)	—	Peng, 2014 [8]
Attenuation of neuroinflammation	—	HBO attenuates M1 microglia/macrophage activation and reduces the proportion of neurotoxic microglia	—	Yang, 2015 [27] Wang, 2019 [10] Wang, 2023 [28]
	—	Pathway: downregulation of TNF‐α; silencing of miR‐204‐5p‐targeted CLCN3; activation of JNK/STAT pathway	—	Yang, 2015 [27] Wang, 2019 [10] Wang, 2023 [28]
Oxidative stress modulation	—	HBO decreases oxidative stress levels and reduces neuronal injury	—	Ostrowski, 2006 [23] Hu, 2017 [31]
	—	Pathway: downregulation of NADPH oxidase activity and gp91^phox^ mRNA; activation of ATP/NAD^+^/Sirt1 pathway	—	Ostrowski, 2006 [23] Hu, 2017 [31]
Cerebral metabolism improvement	HBO and NBO ameliorate derangements in cerebral oxygenation and metabolism	—	—	Zhu, 2015 [32] Nakamura, 2008 [30] Wu, 2024 [37]
Reduction of cerebral edema and BBB disruption	Pathway: prevents occludin degradation and MMP‐9 activation; downregulates HIF‐1α and VEGF (early post‐ICH); downregulates AQP4	Pathway: activates p44/42 MAPK pathway; upregulates RPS6K and HO‐1	Pathway: downregulates NSE and tau protein	Zhou, 2015 [11] Ostrowski, 2005 [25] Qin, 2007 [22] Qin, 2008 [9] Wu, 2014 [33] Li, 2020 [35] You, 2016 [36] Sun, 2010 [39]
Other neuroprotective effects	—	—	NBO exerts neuroprotection by reducing C3‐mediated synaptic pruning	Wu, 2024 [37]
	—	—	Pathway: downregulation of C3	Wu, 2024 [37]

Abbreviations: Akt, protein kinase B; AQP4, aquaporin 4; BBB, blood–brain barrier; C3, complement component 3; CLCN 3, chloride channel protein 3; HBO, hyperbaric hyperoxia; HIF‐α, hypoxia‐inducing factor 1‐α; HO‐1, heme oxygenase‐1; ICH, intracranial hemorrhage; JNK, c‐Jun amino terminal kinase; miR, microRNA; MMP, matrix metalloproteinases; mTOR, mammalian target of rapamycin; NAD^+^, nicotinamide adenine dinucleotide; NBO, normobaric hyperoxia; NSE, neuron specific enolase; PCNA, proliferative cell nuclear antigen; PI3K, phosphatidylinositol 3‐Kinase; RPS6K, ribosomal protein S6 kinases; Sirt1, silent mating type information regulation 2 homolog 1; STAT, signal transduction and activator of transcription; TNF‐α, tumor necrosis factor‐α; VEGF, vascular endothelial growth factor; vWF, von Willebrand factor.

## 4 Effectiveness of Oxygen Therapy in Patients with ICH

### 4.1 Oxygen Therapy in Clinical Trial

Based on patients who had hypertensive intracerebral hemorrhage and were subjected to craniotomy hematoma plus decompressive craniectomy with/without postoperative HBO, Wang et al. compared the effects of HBO on the changes in Glasgow coma scale (GCS) scores and Glasgow outcome scale (GOS) scores of these patients. Significant differences were observed in the GCS and GOS scores at 3 and 5 weeks, which could improve the consciousness and prognosis of patients with ICH after craniotomy [18]. Li et al. conducted a group experiment on patients with acute severe hypertensive basal ganglia hemorrhage after surgery. The length of stay in the hyperbaric oxygen chamber at each exposure session was 100 min for all patients in the five study groups. Four intervention groups receiving HBO (Group B with 2.0 ATA pressure and HBO exposure for 60 sessions; Group C with 2.0 ATA and 90 sessions; Group D with 1.5 ATA and 60 sessions; and Group E with 1.5 ATA and 90 sessions) showed significantly improved modified Barthel index (MBI) and modified Rankin scale (mRS) scores and significantly reduced mortality rates compared with Group A (sham‐control group), indicating that HBO can significantly improve the postoperative survival rate and functional prognosis of patients with cerebral hemorrhage. This study also proposed that a pressure of 1.5 ATA and 60 sessions represents an optimal protocol for HBO [2]. Xu et al. compared the short‐ and long‐term neurological outcomes of patients using the national institutes of health stroke scale (NIHSS), Barthel index (BI), mRS, and GOS. During a 6‐month long‐term follow‐up, patients in the HBO group had a better prognosis than those in the NBO group, and relatively good neurological outcomes were observed. The mRS and MBI scores indicated that patients receiving HBO had a better recovery of their ability to live independently. During long‐term follow‐up, no significant differences in the BI and GOS scores were observed between patients in the HBO and NBO groups; however, significant differences were observed in the mRS and NIHSS scores, indicating that the role of HBO in promoting the recovery of neurological function and the ability to live independently in patients with ICH was stronger than that of NBO, whereas no evident differences were noted in other aspects. The use of HBO therapy at 2.5 ATA is safe, feasible, and effective for treating acute stroke. These two studies have drawn different conclusions regarding the selection of oxygen pressure for HBO, suggesting that an appropriate regimen should be selected according to the patient's condition when using HBO [40].

We compiled the results of clinical trials of oxygen therapy after ICH (Table 2). Compared with patients receiving other treatments, those in the HBO group performed better on multiple indicators. The GCS and NIHSS scores showed that patients receiving HBO had better recovery of neurological function and consciousness. Oxygen therapy has various potential protective effects on the pathophysiological processes after ICH and may be beneficial to patients in terms of neurological function recovery. However, these studies primarily focused on laboratory and small‐scale clinical studies, and the number of studies was relatively small. Large‐scale randomized controlled trials are required to verify their applicability in clinical practice.

**TABLE 2:** Efficacy of oxygen therapy on ICH in clinical trials.

Study	Design	Sample size	Populations	HBO/NBO protocol	Outcomes	Efficacy
Wang, 2020 [18]	Retrospective study	40/41	ICH and underwent clearance of hematoma and decompressive craniectomy	2.0ATA, 60 min, once a day for 20 days	GCS; GOS	Beneficial
Li, 2017 [2]	RCT	105/107	Acute severe ICH	2.0 ATA, 60 min, 60 sessions	MBI test; mRS; mortality rates	Beneficial
		107/107	Acute severe ICH	2.0 ATA, 60 min, 90 sessions	MBI test; mRS; mortality rates	Beneficial
		106/107	Acute severe ICH	1.5 ATA, 60 min, 60 sessions	MBI test; mRS; mortality rates	Beneficial
		108/107	Acute severe ICH	1.5 ATA, 60 min, 90 sessions	MBI test; mRS; mortality rates	Beneficial
Xu, 2018 [40]	RCT	47/32	ICH onset < 24 h	HBO protocol: 2.5 ATA, 60 min, once a day for 30 days; NBO protocol: 1.5 ATA, 60 min, once a day for 30 days	NIHSS; BI; mRS; GOS	Beneficial (better in HBO compared with NBO, evaluated by mRS and NIHSS)

Abbreviations: AIS, acute ischemic stroke; ATA, atmospheres absolute; BI, Barthel index; GCS, Glasgow coma scale; GOS, Glasgow outcome scale; HBO, hyperbaric hyperoxia; ICH, intracranial hemorrhage; MBI, modified Barthel index; mRS, modified Rankin scale; *n*, number patients; NBO, normobaric hyperoxia; NIHSS, National institutes of health stroke scale; RCT, randomized controlled trial; SD, standard deviation; SSS, Scandinavian stroke score.

### 4.2 Promoting Effect of Oxygen Therapy on Neurological Function Recovery and Long‐Term Prognosis

Oxygen therapy has shown clinical advantages in improving the recovery of neurological function and long‐term prognosis in patients with ICH. In patients with moderate‐volume cerebral hemorrhage in the thalamus‐inner capsule region, those treated with stereotactic surgery combined with early HBO had definitive hematoma clearance, shorter illness duration, fewer complications, and better long‐term neurological recovery than those treated with conservative and neuroendoscopic surgical treatments [41]. This result emphasizes the value of oxygen therapy in postoperative rehabilitation, suggesting that early implementation of oxygen therapy may be key to improving the therapeutic effect. Patients with aneurysmal subarachnoid hemorrhage (aSAH) receiving HBO had a lower incidence of delayed cerebral ischemia (DCI) and better improvement in executive control function on the attention network test compared to those without HBO [42]. However, no significant differences were observed in terms of orienting, alerting, mean reaction time, or accuracy between the two groups. HBO has significantly influenced memory function in patients with chronic hemorrhagic stroke. One study analyzed the effects of HBO treatment on memory impairments in poststroke patients during the late chronic, unremitting stage. All memory measures significantly improved after HBO therapy [43]. A case series study has indicated that over 50% of patients with aSAH achieved a good functional prognosis after combined rehabilitation and HBO. The severity of neurological impairment before treatment is closely associated with poor prognosis. The more severe the damage to neurological function, the worse the prognosis [44]. Another study has demonstrated that HBO combined with music therapy substantially improved cerebral neurological deficits, slowed cerebral artery blood flow, promoted the recovery of cerebral function in patients with postoperative aSAH, alleviated anxiety and depression, and improved patients' activities of daily living [45]. Thus, oxygen therapy has a positive effect on mental health and quality of life of patients. Moreover, it is a supplemental treatment that is administered in patients with normal oxygen saturations compared to patients with hypoxia for whom oxygen is required owing to the poorer prognosis associated with hypoxia. In patients with normal oxygen saturations, oxygen therapy is not intended to correct hypoxemia, but rather to improve the oxygenation of ischemic and hypoxic brain tissue surrounding the hematoma. In conclusion, oxygen therapy can promote the recovery of neurological function and provide support during long‐term rehabilitation.

However, the study in the clinical section is dominated by small, single‐center studies, characterized by a low level of evidence and a lack of large, multicenter randomized controlled trials. In addition, differences in oxygen dosage, duration, and timing among studies, as well as the translational challenges between animal findings and human outcomes, underscore the need for large‐scale randomized controlled trials to clarify the optimal treatment regimens and applicable populations.

## 5 Safety of Oxygen Therapy for Patients with ICH

### 5.1 Safety of Oxygen Therapy

Fujiwara et al. tested the safety of NBO in a rat model of intracerebral hemorrhage. NBO did not worsen the hemorrhage severity or brain edema. No significant differences were observed in hemorrhagic or brain water content. NBO did not worsen outcomes in a rat model of ICH. The initial safety data provided suggested that NBO could potentially be started even before a definitive diagnosis is made to distinguish between ischemic and hemorrhagic strokes [46].

Li et al. [2] have reported that patients did not experience severe upper gastrointestinal bleeding owing to HBO treatment and that HBO could be used to treat acute or subacute ICH. Thus, the rational use of oxygen therapy does not increase ICH severity or cause serious adverse reactions. Based on the specific characteristics of patients' conditions, such as the type of hemorrhagic or ischemic cerebrovascular disease, lesion location, severity of the condition, and individual physiological state, the recovery of patients' neurological functions can be safely supported by controlling key elements, such as the treatment timing, treatment frequency, and treatment duration of HBO or NBO [47].

### 5.2 Potential Risks and Controversies of Oxygen Therapy

Although oxygen therapy has shown positive effects on the rehabilitation process of patients with ICH, its potential adverse reactions and risks must be considered. Catalano et al. [48] proposed that excess oxygen therapy is associated with adverse outcomes in patients with nontraumatic SAH. Patients exposed to hyperoxia show poor functional recovery and an increased mortality rate. Hyperoxia may worsen neurological outcomes in such conditions through various mechanisms, such as direct cerebral vasoconstriction or increased excitotoxicity, which in turn leads to lipid peroxidation and the generation of harmful reactive oxygen species [49]. Moreover, hyperoxia exposure is associated with an increased DCI risk and poor outcomes after SAH. Hyperoxia exposure was related to approximately three times the risk of DCI and twice the risk of a poor 3‐month outcome. This relationship was independent of SAH severity and other comorbidities [50]. Thus, during oxygen therapy, the blood oxygen levels of patients should be closely monitored to reduce potential vascular‐related complications.

Another potential risk of oxygen therapy is that it may lead to cerebral vasospasm, exacerbate cerebral ischemic injury, and affect long‐term patient prognosis. Improper oxygen therapy may cause hyperoxemia, which is associated with an increased incidence of cerebral vasospasms in patients with aSAH. This may be because the generation of free radicals increases during hyperoxia, leading to damage to vascular endothelial function, ultimately causing cerebral vasoconstriction [51]. This study revealed the potential risks of hyperoxia in patients with SAH, suggesting that when using oxygen therapy, balancing its protective effects on ischemic tissues and its impact on vascular function is necessary. Some researchers have investigated the effect of hyperoxia on the polarity of AQP4 in an experimental mouse model of intracerebral hemorrhage and reported that hyperoxia regulates AQP4 expression through the osteopontin/MMP‐9/β‐dystroglycan pathway, which may affect the absorption of cerebral edema and BBB stability, thus causing additional damage to brain tissue [52]. This study provides an explanation at the molecular mechanism level, suggesting that hyperoxia may affect the repair process after ICH by altering the expression of key proteins. Therefore, the impact of hyperoxia needs to be carefully evaluated on different pathological states in clinical settings.

Administering NBO and HBO has some practical difficulties. Given the tightness and narrowness of the HBO treatment chamber, patients with ICH need to be treated in the chamber alone for up to 2 h, which is very difficult for some patients with low compliance, especially those with severe conditions. In the hyperacute phase of stroke, the patient's condition is unstable and may progress very rapidly [53]. If a sudden attack occurs during the treatment, the hyperbaric oxygen chamber cannot be abruptly stopped because it involves the process of decompression. In addition, the paralysis rate of ICH is high, and most patients still have sequelae within a short period, requiring medical staff and family members to push them to the HBO treatment cabin, which reflects the inconvenience of HBO [19]. Compared with HBO, NBO has high portability and no restrictions on its place of use, so it may be more suitable for patients with hyperacute stroke, which is a simple and cost‐effective strategy that has the potential to enhance ICH therapy, particularly in resource‐limited settings.

Some studies have explored the role of HBO in postoperative management, believing that it is helpful for postoperative recovery. However, owing to individual differences, individualized treatment plans need to be optimized [54]. This view further supports that oxygen therapy needs to be adjusted according to the specific conditions of the patient to ensure the maximization of curative effects while reducing adverse reactions. Blood oxygen levels must be monitored closely during oxygen therapy to reduce unnecessary risks. In patients with intracerebral hemorrhage admitted to the ICU, Earl and Maharaj [55] calculated the hyperoxemia dose, defined as the area under the arterial partial pressure of oxygen (PaO_2_) time curve above the threshold PaO_2_ value of 100 mmHg (13.3 kPa) divided by the number of hours of potential exposure, and observed an association between the hyperoxemia dose within the first 24 h and ICU mortality. Grensemann et al. [56] have demonstrated a dose‐dependent association between sustained hyperoxia and higher 30‐day mortality as well as reduced favorable functional outcomes at 3 months. A time‐weighted average PaO_2_ range of 78–85 mmHg was associated with the lowest 30‐day mortality and the most favorable outcomes. This is the threshold for PaO_2_ above which toxic effects precipitate. Thus, although hyperoxia has a neuroprotective effect within a certain range, its excessive use may be counterproductive [48]. Yin et al. have reported that after controlling for other factors, data from the MIMIC‐IV database showed that pulse oximetry‐derived oxygen saturation (SpO_2_) was U‐shaped and associated with in‐hospital mortality in patients with SAH. This study provides large‐scale data indicating that in‐hospital mortality was lowest when SpO_2_ was between 94% and 96%. SpO_2_ levels that are excessively high or insufficiently low negatively affects patient survival [57]. After liberal oxygen use in patients with SAH, a higher SpO_2_ (especially a range of minimum SpO_2_ > 97%) is associated with worse outcomes in the most severely ill patients, according to the level of consciousness. The potential harm caused by cumulatively high oxygen does should not be neglected in patients with mild or moderate disease in the general ward [58]. Thus, the oxygen dosage should be carefully adjusted in clinical applications to avoid the potential risks associated with hyperoxia.

The specific impact of oxygen therapy on the prognosis of patients with ICH remains controversial, and different studies have conflicting conclusions. We summarized the results of these existing studies regarding risk thresholds and adverse events in Table 3. When SpO_2_ exceeds a certain threshold, the risk of a poor prognosis for patients increases significantly. This is consistent with the conclusions of previous studies that oxygen therapy has a protective effect within a certain range, although excessive use may lead to oxidative stress, exacerbation of cerebral edema, or abnormal cerebral hemodynamics. Therefore, future research should focus on identifying the ideal SpO_2_ range for ICH. Additionally, when formulating an oxygen therapy plan, the individual differences in patients and changes in their conditions need to be comprehensively considered. Future research should further optimize oxygen therapy plans to achieve a balance between the risks and benefits.

**TABLE 3:** The relationship between the prognosis of oxygen therapy for ICH and blood oxygen levels.

Disease	Blood oxygen monitoring indicator	Optimal range of PaO_2_/SpO_2_	Risk thresholds of PaO_2_/SpO_2_	Adverse event indicator	Study
SAH	PaO_2_	78–85 mmHg	85 mmHg	30‐day mortality and favorable outcome	Grensemann, 2022 [56]
Intracerebral hemorrhage	SpO_2_	ICU cohort: 93%–97%	ICU cohort: 97%	In‐hospital mortality	Zhao, 2024 [58]
		General ward cohort: 95%–97%	General ward cohort: 97%		
SAH	SpO_2_	94%–96%	96%	In‐hospital mortality	Yin, 2022 [57]

*Note:* When the PaO_2_/SpO_2_ integrals remained within the optimal range, patients with ICH exhibited the lowest mortality rate and the highest proportion of favorable outcomes. In contrast, values exceeding the established risk thresholds were dose‐dependently associated with a significant increase in both unfavorable outcomes and mortality among ventilated ICH patients.

Abbreviations: ICU, intensive care unit; PaO_2_, arterial partial pressure of oxygen; SAH, subarachnoid hemorrhage; SpO_2_, Pulse oximetry‐derived oxygen saturation.

## 6 Conclusions

Oxygen therapy treats patients with ICH through multiple mechanisms. HBO reduces cerebral vasospasm, promotes angiogenesis, inhibits inflammation, protects the BBB, reduces brain edema, and improves aerobic metabolism. NBO protects the BBB, reduces brain edema, enhances mitochondrial function, and improves brain metabolism. Clinical trials have indicated that oxygen therapy improves neurological function and long‐term outcomes, and has better therapeutic effects than other treatments in many aspects. However, hyperoxia may increase risks such as DCI and oxidative stress, highlighting the need for controlled oxygen concentration. Future research should further clarify the mechanisms of oxygen therapy for ICH, clarify the optimal oxygen dosage, treatment timing, and long‐term safety of oxygen therapy, optimize its application strategy in patients with ICH, improve its clinical efficacy, and reduce possible adverse reactions.

## Author Contributions

Qiqi Shi prepared the initial manuscript draft. Song Han, Xiangyu Li, Jingwei Guan, Yanli Duan, and Zhangyuan Liao conducted the literature search. Zhiying Chen, Weili Li, Xiangyu Li, Ran Meng, and Ming Zou reviewed and edited the manuscript. Jiayue Ding conceived the study and further contributed to writing, reviewing, and editing the article.

## Funding

This study was sponsored by the National Natural Science Foundation of China (82201432), Natural Science Foundation of Tianjin Municipality (24JCZXJC00320), and Tianjin Key Medical Discipline (Specialty) Construction Project (TJYXZDXK‐3‐003C).

## Ethics Statement

Ethical approval was not required for this review article as it did not involve any original research with human participants or animals.

## Conflicts of Interest

The authors declare no conflicts of interest.

## Data Availability

Data sharing not applicable to this article as no datasets were generated or analyzed during the current study.
